# Muscle Adaptations to High-Load Training and Very Low-Load Training With and Without Blood Flow Restriction

**DOI:** 10.3389/fphys.2018.01448

**Published:** 2018-10-16

**Authors:** Matthew B. Jessee, Samuel L. Buckner, J. Grant Mouser, Kevin T. Mattocks, Scott J. Dankel, Takashi Abe, Zachary W. Bell, John P. Bentley, Jeremy P. Loenneke

**Affiliations:** ^1^School of Kinesiology, University of Southern Mississippi, Hattiesburg, MS, United States; ^2^Exercise Science Program, University of South Florida, Tampa, FL, United States; ^3^Department of Kinesiology and Health Promotion, Troy University, Troy, AL, United States; ^4^Department of Exercise Science, Lindenwood University – Belleville, Belleville, IL, United States; ^5^Kevser Ermin Applied Physiology Laboratory, Department of Health, Exercise Science, and Recreation Management, The University of Mississippi, Oxford, MS, United States; ^6^Department of Pharmacy Administration, The University of Mississippi, Oxford, MS, United States

**Keywords:** ischemia, resistance training, volitional failure, kaatsu, exercise

## Abstract

An inability to lift loads great enough to disrupt muscular blood flow may impair the ability to fatigue muscles, compromising the hypertrophic response. It is unknown what level of blood flow restriction (BFR) pressure, if any, is necessary to reach failure at very low-loads [i.e., 15% one-repetition maximum (1RM)]. The purpose of this study was to investigate muscular adaptations following resistance training with a very low-load alone (15/0), with moderate BFR (15/40), or with high BFR (15/80), and compare them to traditional high-load (70/0) resistance training. Using a within/between subject design, healthy young participants (*n* = 40) performed four sets of unilateral knee extension to failure (up to 90 repetitions/set), twice per week for 8 weeks. Data presented as mean change (95% CI). There was a condition by time interaction for 1RM (*p* < 0.001), which increased for 70/0 [3.15 (2.04,4.25) kg] only. A condition by time interaction (*p* = 0.028) revealed greater changes in endurance for 15/80 [6 (4,8) repetitions] compared to 15/0 [4 (2,6) repetitions] and 70/0 [4 (2,5) repetitions]. There was a main effect of time for isometric MVC [change = 10.51 (3.87,17.16) Nm, *p* = 0.002] and isokinetic MVC at 180^°^/s [change = 8.61 (5.54,11.68) Nm, *p* < 0.001], however there was no change in isokinetic MVC at 60^°^/s [2.45 (−1.84,6.74) Nm, *p* = 0.261]. Anterior and lateral muscle thickness was assessed at 30, 40, 50, and 60% of the upper leg. There was no condition by time interaction for muscle thickness sites (all *p* ≥ 0.313). There was a main effect of time for all sites, with increases over time (all *p* < 0.001). With the exception of the 30% lateral site (*p* = 0.059) there was also a main effect of condition (all *p* < 0.001). Generally, 70/0 was greater. Average weekly volume increased for all conditions across the 8 weeks, and was greatest for 70/0 followed by 15/0, 15/40, then 15/80. With the exception of 1RM, changes in strength and muscle size were similar regardless of load or restriction. The workload required to elicit these changes lowered with increased BFR pressure. These findings may be pertinent to rehabilitative settings, future research, and program design.

## Introduction

Studies have shown that training with lower loads (i.e., 30% 1RM) to volitional failure elicits increases in muscle size and strength similar to high-load resistance training (Mitchell et al., 2012; Morton et al., 2016). However, there may be a point at which the external training load is too low to fully stimulate muscular adaptations. A 12-week training protocol comparing 70% 1RM and 15.5% 1RM, found muscle size and strength adaptations favored the high-load condition (Holm et al., 2008). The results, however, may be limited by the methodology of matching exercise volume (i.e., total kg lifted; reps × load) in the 15.5% 1RM to that completed during 70% 1RM. While exercise to failure is not always necessary for adaptation, it has been suggested as a more appropriate strategy to truly compare the efficacy of exercise protocols (Dankel et al., 2016).

During dynamic exercise, the ability to reach volitional failure at the muscular level may depend on generating a contraction strong enough to disrupt muscle blood flow. At very-low isometric contraction intensities the changes in mean arterial pressure are small and may lead to a prolonged contraction time (Hunter and Enoka, 2001). If the exercise load is too low and does not induce some level of fatigue the muscular size and strength adaptation may be attenuated, or may be more aerobic in nature, evidenced by a greater acute mitochondrial protein synthetic response vs. myofibrillar (Burd et al., 2012). As Holm et al. (2008) did not train to failure, it is currently unknown if doing so with a very low-load can stimulate similar muscular adaptations when compared to a traditional high-load protocol.

Applying blood flow restriction (BFR) to the limb is a strategy used to disrupt muscular blood flow and increase fatigability of the muscle during low-load exercise (Ganesan et al., 2015). Over a training program, the addition of BFR significantly reduced the workload required to reach volitional failure, while eliciting similar muscular adaptations compared to non-restricted training at the same relative load (Fahs et al., 2015; Farup et al., 2015). However, the minimum level of pressure necessary for maximal adaptation is unclear: using 30% 1RM Counts et al. (2016) found no difference in muscle hypertrophy between training with high [i.e., 90% arterial occlusion pressure (AOP)] or moderate pressures (40% AOP), whereas Lixandrao et al. (2015) found a greater hypertrophic effect when using a high pressure (i.e., 80% AOP) in conjunction with 20% 1RM, albeit when not exercising to failure. Thus, suggesting a higher pressure (80% AOP) may be necessary when using loads lower than 30% 1RM.

The purpose of the current study was to compare muscle size, strength, and endurance adaptations between high-load and very-low load training to volitional failure. In addition, we sought to determine if applying BFR was necessary to induce adaptation to very low-load training, and if the effect of BFR was pressure dependent. As very low-load exercise may be necessary and/or preferable for certain populations, the findings of this study would give more insight into program design, providing more understanding of the required stimuli (i.e., loading thresholds and restriction application) for muscle size and strength increases. We hypothesized that: both strength and muscle size increases would be augmented in a pressure dependent manner within the 15% 1RM conditions, strength increases would be greatest with 70% 1RM, the 80% BFR condition would be needed to increase muscle size similar to 70% 1RM, and endurance would be greater in all 15% 1RM conditions compared to 70% 1RM.

## Materials and Methods

### Participants

Forty-six participants, between the ages of 18–35 years, were recruited to participate in the study. Participants were untrained and had not engaged in resistance exercise within 6 months prior to beginning the study. Participants were excluded from the study if: they regularly used tobacco products within the previous 6 months, had a BMI ≥ 30 kg/m^2^, an orthopedic injury preventing exercise, or took medication for hypertension. Although it has been shown to be relatively safe, some concern regarding the risk of thromboembolism exists regarding BFR exercise. Thus, participants were also excluded if they met at least two of the following risk factors for thromboembolism: diagnosed with Crohn’s disease, past fracture of the hip, pelvis, or femur, major surgery within the last 6 months, varicose veins, family or personal history of deep vein thrombosis, family or personal history of pulmonary embolism (Motykie et al., 2000). Four participants dropped out of the study prior to beginning training, while two others dropped out during the training period due to personal reasons. No adverse responses to training were observed or reported. The data were analyzed and presented for the 20 males and 20 females completing all visits (with the exception of one individual who missed one training session). This study was approved by The University of Mississippi’s Institutional Review Board. All participants gave written informed consent in accordance with the Declaration of Helsinki.

### Experimental Design

Over the course of 22 total visits, spanning 10 weeks, muscle size, strength, and endurance of the knee extensors were measured before and after an 8-week unilateral knee extension training protocol. Participants trained with two of four possible conditions, one assigned to each leg. The conditions, labeled as % 1RM/% AOP, were: 70/0, 15/0, 15/40, and 15/80. They were assigned in a randomized, counter-balanced fashion, with no participant receiving the same condition in both legs. On the initial pre-visit, if the participant met inclusion criteria, they proceeded to sign a written informed consent document and PAR-Q, then had height and body mass assessed, followed by muscle thickness measurements of both legs. On the second pre-visit, participants were familiarized with procedures to be used for testing knee extension 1RM as well as isometric and isokinetic strength. On the third pre-visit, participants completed knee extension 1RM tests for each leg, then completed isokinetic and isometric strength tests followed by muscular endurance assessment. On visits 4–19 participants completed the 8-week training protocol, training twice per week with a minimum of 24 h separating each visit. Post measurements were taken over three separate days, at least 48 h following the last training session, and resembled pre-training test procedures. Additional measures included a mid-point assessment of muscle thickness, and an assessment of the acute exercise-induced swelling response during training sessions 1, 9, and 15. Of note: the current experiment presented herein was part of a larger training program that also included an upper body training protocol (upper body data reported elsewhere).

### Muscle Thickness

Muscle thickness was measured using B-mode ultrasound (Logiq-e GE, Fairfield, CT, United States) before training, prior to training session 9 (mid-training), and 48 + h after the last training session. While the participant was standing, feet shoulder width apart and weight evenly balanced, a linear array probe (L4-12t GE, Fairfield, CT, United States) was coated with transmission gel and placed against the skin perpendicular to the femur, with care taken not to depress the dermal surface. Two images were saved and stored for each site on the anterior and lateral portion of both legs (30, 40, 50, and 60% of the distance from the greater trochanter to the lateral condyle of the femur). To include an internal measurement control, an additional image was taken on the participant’s left posterior upper arm midway from the acromion process to the olecranon process. Muscle thickness was determined as the average distance between the muscle-bone and muscle-adipose interfaces, assessed to the nearest 0.01 cm, from the two stored images. All measurements and analyses of muscle thickness were taken by the same investigator throughout the study. To limit any bias, the investigator was blinded to each condition during image analysis, which was done only after all testing was completed.

### One-Repetition Maximum

1RM was used as a strength outcome and to determine training loads. 1RM was assessed by finding the greatest load participants could lift one time, with proper form, through a full range of motion using a unilateral knee extension machine (Hammer Strength Iso-Lateral Leg Extension Life Fitness, Rosemont, IL, United States). Prior to testing, as a warmup, a self-determined number of unloaded repetitions were completed, followed by 1 repetition each of an estimated 30 and 70% 1RM. For testing, participants were asked to move a given load from a starting position (knee angle of approximately 90°) to full knee extension one time per attempt, while buckled into the seat with arms crossed over their chest. In an effort to reduce subjectivity, a bar was placed at the top of the range of motion and for the attempt to be classified as successful, the load had to reach the bar. The load was increased following each successful attempt. If unsuccessful, the load was decreased and this process continued until the maximum load the participant could successfully lift was determined. The amount of weight added or removed after each attempt was based upon the speed and effort from the previous attempt. Attempts for each leg were alternated and at least 45 s of rest was observed between attempts (90 s between attempts using the same leg). All testing was supervised by trained personnel.

### Isometric and Isokinetic Strength

Isometric and isokinetic strength were tested using a dynamometer (Quickset System 4 Biodex, Shirley, NY, United States). Prior to all testing, chair and leg attachments were adjusted to properly fit each individual, then settings were recorded to ensure the same testing conditions for all measures. Isokinetic testing was performed at two speeds, 180°/s and 60°/s. While seated, participants performed 2 sets (separated by 60 s rest) of 3 maximal knee extensions (knee angle from approximately 90° to 180°) at 180°/s then at 60°/s. During isometric testing, participants completed two maximal knee extensions with the knee positioned at approximately 90° of flexion. Participants completed two attempts with 60 s rest. Attempts were given up to 15 s, but were stopped prior if a clear decrease or plateau in torque was observed. This resulted in most attempts lasting approximately 3 – 8 s. All testing was performed with participants’ arms crossed over their chest. Participants were also provided with visual feedback and strong verbal encouragement during each attempt. Regarding test order, isokinetic testing was always completed prior to isometric and all three tests were completed on one leg first (randomized), followed by the contralateral leg.

### Endurance

To compare changes in muscular endurance between conditions, participants were asked to complete one set of unilateral knee extension exercise to volitional failure before and after training. The load for pre and post endurance tests was 42.5% of the participants’ pre-training 1RM value as this load was exactly halfway between 15 and 70% 1RM. This relative load was chosen to avoid favoring one loading condition over the other. Prior to testing the seat was adjusted and recorded so that all testing conditions were similar. A lap belt was pulled snuggly across participants’ waist and they were instructed to maintain arms crossed over their chest while the test was being conducted. Participants were instructed to lift the load from the starting position until touching a bar set at the top of the range of motion for a repetition to be deemed successful. Repetitions were performed at a cadence of 2 s per contraction (1 s concentric and 1 s eccentric). If a participant was unable to complete a full range of motion or maintain proper cadence, the test was terminated. A 5 min rest period was observed between tests.

### Arterial Occlusion Pressure

To apply a relative pressure during 15/40 and 15/80 conditions, AOP was taken prior to exercise while the participant was seated in a knee extension machine. A 10 cm wide nylon cuff (SC10 Hokanson, Bellevue, WA, United States) was applied to the proximal portion of the thigh. An auditory signal of a pulse was found at the posterior tibialis artery using a Doppler probe (MD6 Hokanson, Bellevue, WA, United States). Starting at 50 mmHg the cuff was slowly inflated (E20 Rapid Cuff Inflator Hokanson, Bellevue, WA, United States) until the pulse distal to the cuff was no longer detected. The inflation pressure of the cuff was recorded as AOP and the assigned percentage (40 or 80%) of this pressure was applied during exercise.

### Training Protocol

The 8-week training protocol required 2 supervised training sessions per week, consisting of 4 sets of unilateral knee extensions to volitional failure under the assigned condition. Both legs trained each day with the leg training first alternated between days. Participants were given a self-determined rest period between training each leg. The very low-load conditions (15/0, 15/40, and 15/80) trained with a load equal to 15% of 1RM and had inter-set rest periods of 30 s. The high-load condition (70/0) trained with a load equal to 70% 1RM with 90 s inter-set rest periods. Applied pressure during the BFR conditions [15/40 (40% AOP) and 15/80 (80% AOP)] was set as a percentage of pre-exercise AOP measured each session while participants were in an upright seated position. A 10 cm wide inelastic cuff (SC10 Hokanson, Bellevue, WA, United States) was applied to the most proximal portion of the leg, inflated (E20 Rapid Cuff Inflator Hokanson, Bellevue, WA, United States) prior to exercise, and remained inflated until the cessation of the last set, after which it was deflated and removed. All repetitions were performed to a metronome (1 s concentric and 1 s eccentric). All sets, regardless of condition were ceased at 90 repetitions, as this would equal the time-frame used by Holm et al. (2008), and it would minimize participant strain. Further, if the contractions were not generating a sufficient amount of fatigue the stimulus would likely become more aerobic with time (Burd et al., 2012). To minimize any confounding effects of load on failure, loads were not progressed across training. To minimize soreness associated with novel exercise, sets were ramped at the beginning of training (i.e., training session one, participants completed one set of exercise, another set was added for session two, three sets were completed for sessions three and four, and thereafter four sets of exercise were completed for all remaining sessions).

### Exercise-Induced Swelling

Measures of the acute exercise-induced swelling response were assessed at training sessions 1, 9, and 15 to better determine if chronic changes in muscle thickness were due to swelling rather than muscle growth as a muscle’s ability to swell provides some indication that there is not a large presence of swelling at baseline (Buckner et al., 2017a). The anterior 50% site was measured immediately before and after the exercise protocol using procedures similar to those of muscle thickness except images were frozen and muscle thickness measured immediately using on-screen calipers. Two images were analyzed and an average of the two measures was determined to be muscle thickness. If the two initial measures differed by greater than 0.1 cm, a third image was taken and an average of the two closest measures was used.

### Statistical Analyses

SPSS version 24.0 (SPSS Inc., Armonk, NY, United States) was used for data analysis. To examine changes in all strength, muscle thickness, and exercise volume values across time between groups, while accounting for our within/between subject design, two-factor (condition × time) analysis of variance was used. Special consideration was taken to account for the dependency created because each participant contributed observations in two of the four possible training conditions and at multiple time points. ANOVA models were estimated using covariance pattern models. Two different error covariance structures were compared prior to hypothesis testing: compound symmetry and unstructured. Akaike’s Information Criterion (AIC) and Schwarz’s Bayesian Criterion (BIC) values were compared to determine the most appropriate model. If there was a significant time x condition interaction (*p* ≤ 0.05), we examined simple effects. Otherwise, main effects of time and condition were examined. A one-factor analysis of variance (ANOVA) was used to detect differences across time for control muscle thickness. Results are presented as mean (SE) unless otherwise stated.

## Results

### Demographics

At baseline, participants (*n* = 40) had a mean (SD) age of 21 (2) years, height of 171.56 (9.32) cm, body mass of 68.37 (11.49) kg, and BMI of 23.14 (2.83) kg/m^2^.

### Muscle Thickness

**FIGURE 1:**
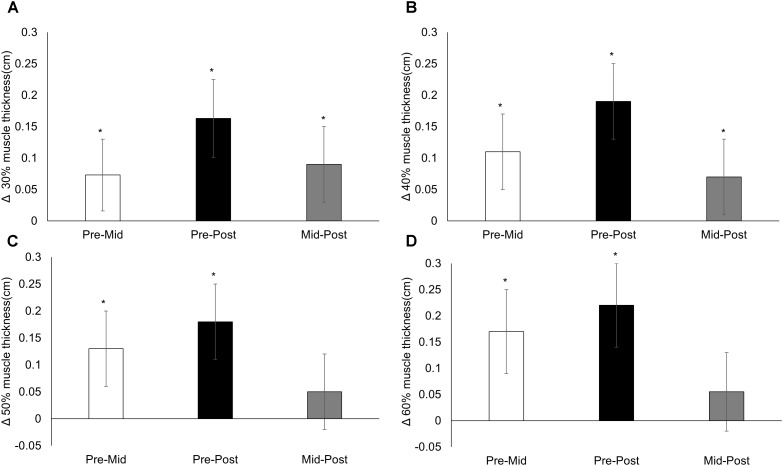
Anterior muscle thickness. Changes from pre-training, mid-training, and post-training for anterior muscle thickness at **(A)** 30%, **(B)** 40%, **(C)** 50%, and **(D)** 60% of the upper leg, collapsed across conditions. Data presented as mean changes (95% CI). An ^∗^ indicates a significant change from pre. Alpha level = 0.05.

**FIGURE 2:**
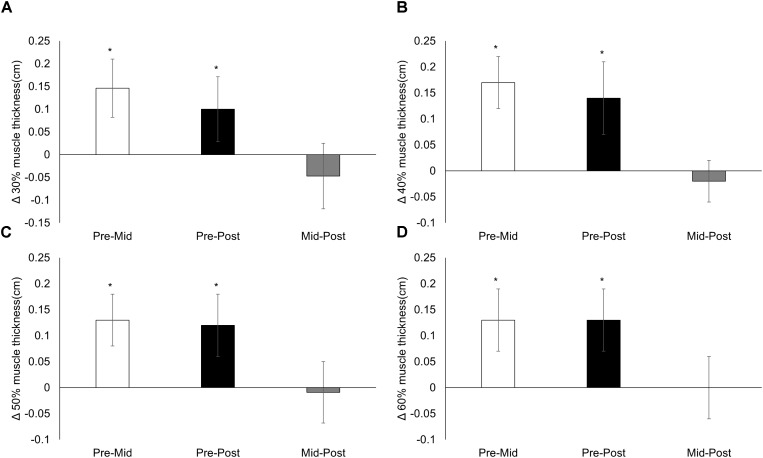
Lateral muscle thickness. Changes from pre-training, mid-training, and post-training for lateral muscle thickness at **(A)** 30%, **(B)** 40%, **(C)** 50%, and **(D)** 60% of the upper leg, collapsed across conditions. Data presented as mean changes (95% CI). An ^∗^ indicates a significant change from pre. Alpha level = 0.05.

There was no difference (mean, 95% CI) across time for control muscle thickness [0.07 (−0.006, 0.151); *p* = 0.054]. There were no time × condition interactions for anterior (30%, *p* = 0.607; 40%, *p* = 0.828; 50%, *p* = 0.782; 60%, *p* = 0.740) or lateral (30%, *p* = 0.492; 40%, *p* = 0.656; 50%, *p* = 0.414; 60%, *p* = 0.354) muscle thickness sites. There was a main effect of time for all anterior (all *p* < 0.001; **Figure 1**) and lateral (all *p* < 0.001; **Figure 2**) muscle thickness sites, which increased in response to training. There was also a main effect of condition for all sites (70/0 greater than all other conditions, all *p* ≤ 0.007) except the 30% lateral site (*p* = 0.058). Changes in anterior and lateral muscle thickness separated by condition can be seen in **Tables 1**, **2**, respectively.

**Table 1:** Anterior muscle thickness changes per condition (cm).

Site	Change	15/0	15/40	15/80	70/0
30%	pre-mid^∗^	0.11 (−0.01, 0.22)	0.00 (−0.13, 0.12)	0.08 (−0.04, 0.20)	0.10 (−0.01, 0.23)
	pre-post^∗^	0.19 (0.07, 0.31)	0.05 (−0.07, 0.18)	0.17 (0.05, 0.30)	0.22 (0.09, 0.34)
40%	pre-mid^∗^	0.13 (0.01, 0.26)	0.11 (−0.01, 0.24)	0.04 (−0.08, 0.17)	0.16 (0.03, 0.29)
	pre-post^∗^	0.20 (0.08, 0.32)	0.14 (0.01, 0.27)	0.19 (0.06, 0.32)	0.22 (0.09, 0.35)
50%	pre-mid^∗^	0.16 (0.03, 0.29)	0.17 (0.04, 0.31)	0.03 (−0.09, 0.17)	0.16 (0.02, 0.29)
	pre-post^∗^	0.19 (0.06, 0.32)	0.18 (0.05, 0.32)	0.17 (0.04, 0.31)	0.20 (0.06, 0.33)
60%	pre-mid^∗^	0.22 (0.07, 0.37)	0.19 (0.04, 0.35)	0.05 (−0.10, 0.21)	0.21 (0.05, 0.37)
	pre-post^∗^	0.23 (0.08, 0.38)	0.21 (0.05, 0.37)	0.20 (0.04, 0.36)	0.25 (0.09, 0.41)

*Changes (cm) in muscle thickness from pre to mid-training and pre to post-training for anterior sites on the upper leg separated by condition. Data presented as mean change (95% CI). An asterisk next to change label indicates a main effect of time, whereby all conditions changed similarly within the respective time period. There were no significant changes between conditions within each time frame. Alpha level = 0.05.*

**Table 2:** Lateral muscle thickness changes per condition (cm).

Site	Time	15/0	15/40	15/80	70/0
30%	pre-mid^∗^	0.10 (−0.03, 0.24)	0.04 (−0.10, 0.18)	0.22 (0.08, 0.37)	0.20 (0.06, 0.35)
	pre-post^∗^	0.10 (−0.02, 0.24)	0.02 (−0.12, 0.17)	0.17 (0.03, 0.32)	0.08 (−0.05, 0.23)
40%	pre-mid^∗^	0.19 (0.11, 0.28)	0.16 (0.07, 0.25)	0.10 (0.02, 0.19)	0.21 (0.12, 0.30)
	pre-post^∗^	0.16 (0.07, 0.26)	0.13 (0.03, 0.23)	0.09 (−0.00, 0.19)	0.19 (0.09, 0.29)
50%	pre-mid^∗^	0.08 (−0.02, 0.20)	0.17 (0.05, 0.29)	0.03 (−0.08, 0.15)	0.22 (0.10, 0.34)
	pre-post^∗^	0.12 (0.00, 0.23)	0.12 (0.00, 0.24)	0.07 (−0.04, 0.19)	0.15 (0.03, 0.27)
60%	pre-mid^∗^	0.13 (0.01, 0.24)	0.22 (0.09, 0.34)	0.01 (−0.10, 0.13)	0.17 (0.05, 0.29)
	pre-post^∗^	0.10 (−0.01, 0.21)	0.18 (0.06, 0.30)	0.10 (−0.02, 0.22)	0.15 (0.03, 0.27)

*Changes (cm) in muscle thickness from pre to mid-training and pre to post-training for lateral sites on the upper leg separated by condition. Data presented as mean change (95% CI). An asterisk next to change label indicates a main effect of time, whereby all conditions changed similarly within that time period. There were no significant changes between conditions within each time frame. Alpha level = 0.05.*

### One-Repetition Maximum

**FIGURE 3:**
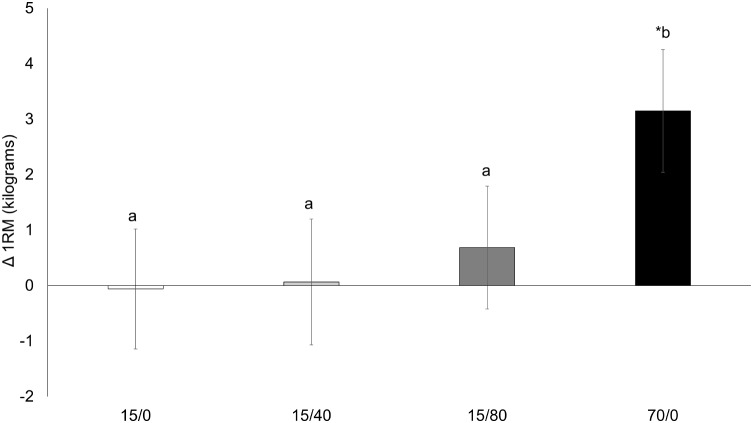
Knee extension 1RM. Changes from pre-training to post-training knee extension 1RM (one-repetition maximum) for each condition. Data presented as mean changes (95% CI). An ^∗^ indicates a significant change from pre. Letters indicate significant differences between conditions. If conditions share the same letter they are not different from one another. Alpha level = 0.05.

A time x condition interaction for 1RM (*p* < 0.001; **Figure 3**) showed the response to training was greater in 70/0 compared to 15/0 [mean difference = 3.2 (0.7) kg, *p* < 0.001], 15/40 [mean difference = 3.0 (0.7) kg, *p* < 0.001], and 15/80 [mean difference = 2.4 kg (0.7), *p* = 0.002]. 70/0 increased 1RM from baseline [29.4 (1.3) to 32.6 (1.3) kg, *p* < 0.001], while 15/0 [30.3 (1.3) to 30.2 (1.3) kg, *p* = 0.913], 15/40 [30.0 (1.3) to 30.1 (1.3) kg, *p* = 0.909), and 15/80 [28.6 (1.3) to 29.3 (1.3) kg, *p* = 0.220] did not.

### Isometric and Isokinetic Strength

**FIGURE 4:**
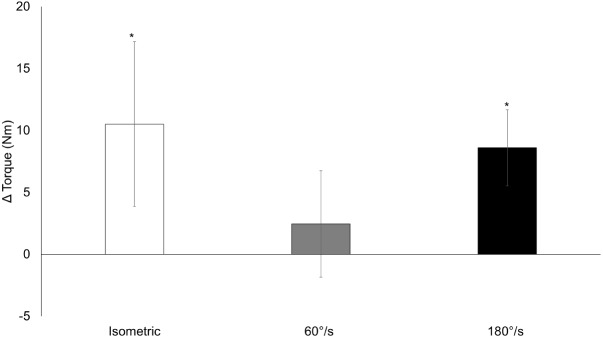
Isometric and isokinetic torque. Changes from pre-training to post-training for isometric and isokinetic strength at 60°/s and 180°/s collapsed across conditions. Data presented as mean changes (95% CI). An ^∗^ indicates a significant change from pre. Alpha level = 0.05.

There was no time x condition interaction for isometric strength (*p* = 0.292) or isokinetic strength at 60°/s (*p* = 0.537) and 180°/s (*p* = 0.180). There was a main effect of time for isometric strength (219.5 (10.3) to 230.1 (10.3) Nm, *p* = 0.002) and isokinetic strength at 180°/s [139.2 (6.8) to 147.8 (6.8) Nm, *p* < 0.001], while isokinetic strength at 60°/s did not change [198.0 (8.0) to 200.5 (8.0) Nm, *p* = 0.261]. Changes for dynamometry are depicted in **Figure 4** and can be seen separated by condition in **Table 3**.

**Table 3:** Isometric and isokinetic strength changes per condition (Nm).

MVC	15/0	15/40	15/80	70/0
Isometric^∗^	−0.50 (−13.45, 12.45)	13.99 (0.37, 27.61)	13.15 (−0.12, 26.43)	15.42 (2.14, 28.70)
60°/s	−2.01 (−10.39, 6.37)	3.99 (−4.81, 12.80)	1.29 (−7.29, 9.88)	6.52 (−2.06, 15.11)
180°/s^∗^	6.92 (0.91, 12.93)	5.94 (−0.38, 12.28)	7.07 (0.91, 13.24)	14.51 (8.45, 20.57)

*Changes (Nm) from pre to post-training in isometric and isokinetic (60°/s and 180°/s) maximal voluntary contraction (MVC) separated by condition. Data presented as mean change (95% CI). An asterisk next to MVC label indicates a main effect of time, whereby all conditions changed similarly from baseline. There were no significant differences between conditions for any measure. Alpha level = 0.05.*

### Endurance

**FIGURE 5:**
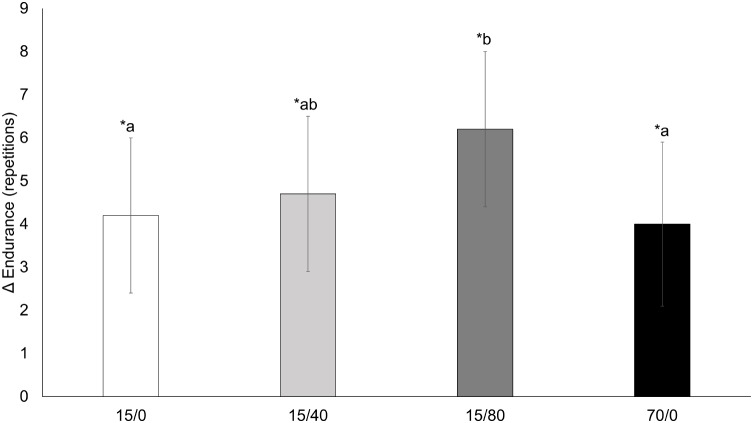
Knee extension endurance. Changes from pre-training to post-training knee extension endurance repetitions for each condition. Data presented as mean changes (95% CI). An ^∗^ indicates a significant change from pre. Letters indicate significant differences between conditions. If conditions share the same letter they are not different from one another. Alpha level = 0.05.

A time x condition interaction for endurance repetitions (*p* = 0.028; **Figure 5**) showed the increase in repetitions for 15/80 was greater compared to 15/0 [mean difference = 1.9 (0.7) repetitions, *p* = 0.014] and 70/0 [mean difference = 2.1 (0.7) repetitions, *p* = 0.006]. Endurance repetitions increased for all conditions: 15/0 = 20 (1.1) to 24 (0.9) repetitions, *p* < 0.001; 15/40 = 21 (1.1) to 25 (1.0) repetitions, *p* < 0.001; 15/80 = 21 (1.1) to 27 (0.9) repetitions, *p* < 0.001; 70/0 = 22 (1.1) to 26 (0.9) repetitions, *p* < 0.001.

### Exercise-Induced Swelling

**FIGURE 6:**
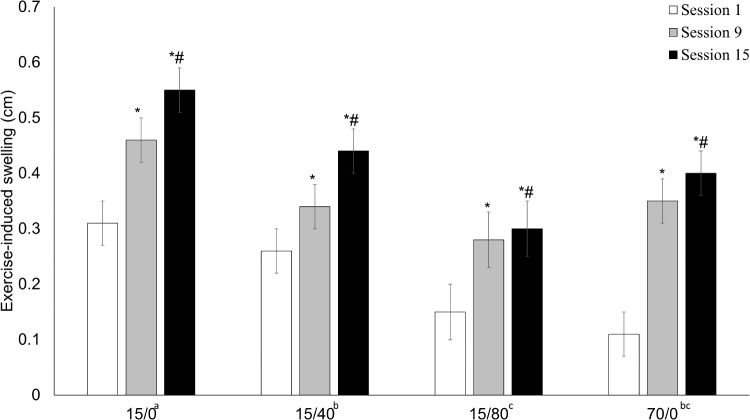
Exercise-induced swelling response. The exercise-induced swelling response to session 1, session 9, and session 15. Data presented as mean (SE). An ^∗^ indicates a significant difference from session 1, whereas # indicates a significant difference from session 9. Letters next to condition labels indicate significant differences (main effect of condition). If conditions share the same letter they are not different from one another. Alpha level = 0.05.

There was no time x condition interaction for the muscle swelling response (*p* = 0.574). There was, however, a main effect of time (*p* < 0.001; **Figure 6**) and condition (*p* < 0.001). For all conditions, the muscle swelling response increased from training session 1 to session 9 [0.14 (0.03) cm, *p* < 0.001] and again from session 9 to session 15 [0.06 (0.03) cm, *p* = 0.042). Collapsed across time, 15/0 elicited the greatest swelling response (all *p* ≤ 0.014), and 15/40 was greater than 15/80 (*p* = 0.011).

### Exercise Volume

**FIGURE 7:**
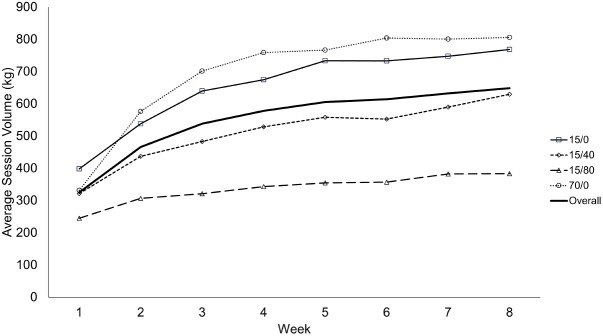
Average training volume per session during each week of the training protocol. Data presented as mean values. This figure is a visual aid, and as such, no statistical comparisons are denoted.

Weekly exercise volume was calculated as the average number of repetitions completed over the two weekly training sessions, multiplied by the load lifted. There was a time x condition interaction for weekly exercise volume (*p* < 0.001; **Table 4**). In general, volume increased in most successive weeks, for each condition, throughout the training protocol. In week 1, 15/40, and 70/0 did not differ in volume (*p* = 0.365) nor did 15/0 and 70/0 in weeks 2, 5, 6, 7, and 8 (all *p* ≥ 0.183). All other conditions differed from one another when comparing them in each remaining week (all *p* ≤ 0.021). Two participants, using condition 15/0, reached 360 goal repetitions, one at session 14 the other at session 5. Both completed all repetitions for the remaining sessions (**Figure 7**). All others reached volitional failure at some point during the protocol.

**Table 4:** Average weekly exercise volume per session (kg).

Week	15/0	15/40	15/80	70/0	Condition
1	403.2^a^	315.2^a^	214.2^a^	329.3^a^	15/0 vs. all; 15/80 vs. all
2	533.5^b^	436.1^b^	278.2^b^	568.8^b^	15/0 vs. 15/40, 15/80; 15/40 vs. all; 15/80 vs. all
3	637.2^c^	492.3^c^	298.9^b^	704.5^c^	15/0 vs. all; 15/40 vs. all; 15/80 vs. all
4	676.4^d^	537.1^d^	320.7^c^	762.8^de^	15/0 vs. all; 15/40 vs. all; 15/80 vs. all
5	732.2^e^	582.1^ef^	343.4^de^	756.6^d^	15/0 vs. 15/40, 15/80; 15/40 vs. all; 15/80 vs. all
6	745.8^e^	571.5^e^	339.3^cd^	790.7^ef^	15/0 vs. 15/40, 15/80; 15/40 vs. all; 15/80 vs. all
7	764.1^f^	610.5^f^	367.1^e^	803.8^fg^	15/0 vs. 15/40, 15/80; 15/40 vs. all; 15/80 vs. all
8	799.2^g^	611.0^f^	365.2^de^	825.9^g^	15/0 vs. 15/40, 15/80; 15/40 vs. all; 15/80 vs. all

*Average training volume (kg) per session during each week (1–8) of the training protocol. Data presented as mean. Letters indicate significant differences between weeks within each condition. If at least one letter is similar there are no differences between weeks. Condition column indicates significant differences between listed conditions within each week. If not listed conditions are not different from each other. Alpha level = 0.05.*

## Discussion

The main findings of the current study were that 1RM changes favored the high-load condition, while isometric and isokinetic strength responded similarly across all conditions. The increase in endurance was greater in the 15/80 condition compared to 15/0 and 70/0. The increase in muscle thickness from training seemed to be uniform across conditions. We believe the changes in muscle thickness were due to muscle growth rather than edema as there was an exercise-induced swelling response at training sessions 1, 9, and 15, suggesting minimal swelling prior to exercise at each phase of training. Training volume was greatest in non-restriction conditions and then decreased with increased pressure.

### Strength

When comparing strength outcomes to Holm et al. (2008) who also compared 15 and 70% 1RM, and a recent meta-analysis by Lixandrão et al. (2018) we found similar results in that changes in 1RM favor high-loads over low-loads. However, we diverge from Holm et al. (2008) regarding our low-load conditions, which did not increase 1RM in the current study. The responses in 1RM, favoring high-load over very low-load, were expected due to the practicing of a skill that more closely resembles this specific strength test (Buckner et al., 2017b). To illustrate, previous studies have found that changes in 1RM favor high-load training over low-loads (Mitchell et al., 2012; Lasevicius et al., 2018), yet when a low-load group periodically practices the 1RM test the differences are diminished (Morton et al., 2016). During the study by Holm et al. (2008), 1RM was assessed (practiced) every 10th training session, which may explain the difference from our low-load conditions as our participants were only tested pre and post, thus having minimal practice of the test.

We found no effect of load or BFR on dynamometry measured strength changes while Holm et al. (2008) found a favorable change for high loads. BFR has been previously shown to augment the isometric (Shinohara et al., 1998) and 1RM (Laurentino et al., 2012) strength response to low-load training, but that was not observed in the current study. Kacin and Strazar (2011) also found no effect of very low-load training (15% maximum strength) with or without restriction on isometric strength changes. In comparison with previous studies that used 20% 1RM (Laurentino et al., 2012) and 40% (Shinohara et al., 1998) maximum strength respectively, training with 15% 1RM, regardless of restriction, may be too low to induce a meaningful increase in maximal isometric or isokinetic strength. This suggests either a potential loading threshold, or a lack of practice with the test overall as we only performed dynamometry pre and post-training. While we did find a time effect for improvements in isometric and isokinetic strength at 180°/s, they were small relative to the movement and the population tested (young healthy adults). Future research should seek to compare the response to these conditions in clinical populations to determine the effect on muscle strength. While the relative improvement in this study was low, the effect could be greater or more meaningful in those with limited physical function. Further if BFR reduces the workload while providing similar muscular improvements it could be an effective therapeutic tool.

### Endurance

While endurance improved across all conditions it was augmented over 15/0 and 70/0 by combining a very low-load with BFR at 80% AOP. While Holm et al. (2008) did not measure endurance as an outcome, Kacin and Strazar (2011) found that BFR training augmented endurance over an equally loaded, non-restricted group. The mechanism causing this greater response to endurance may either be strictly physiological, psychophysiological, or both. During resistance exercise with 20% 1RM, greater BFR pressure (i.e., 230 mmHg) augments the metabolic stress compared to BFR with 180 mmHg and a non-restriction condition (Sugaya et al., 2011). BFR also augments angiogenic gene expression in response to acute low-load resistance exercise (Larkin et al., 2012; Ferguson et al., 2018). Over a chronic training period the adaptation to 15/80 may have reflected the greater metabolic disturbances and angiogenic gene expression within the muscle, thus increasing the capacity to deal with a metabolic disturbance and/or result in a greater capillarization compared to 15/0 and 70/0. In addition, psychophysiological adaptations may have also occurred. For example, as greater BFR pressures (i.e., 80% AOP) are associated with greater perceptions of exertion and discomfort at very low-loads (Jessee et al., 2017; Dankel et al., 2018), the participants training with 80% AOP may have become accustomed to these feelings and more able to withstand similar feelings during an endurance test to volitional failure, resulting in more repetitions. The increased endurance from pre to post-training in all conditions is supported by the increased weekly volume during training which could reflect an increased work capacity. Although the exact mechanism was not explored in the current study, it seems as though greater BFR pressure (i.e., 80% AOP) augments the improvement in endurance over very low loads alone. This finding could have positive implications for clinical and elderly populations as activities of daily living are often submaximal and repetitive; however, future research should examine if these improvements in knee extension endurance do indeed transfer to activities of daily living.

### Muscle Thickness and Swelling

Our findings of similar improvements in muscle thickness across all sites, regardless of condition, differ from those of Holm et al. (2008) and Lixandrao et al. (2015). We believe the discrepancies can be explained by exercising to volitional failure. While on average our participants exercised to volitional failure (only 2 participants reached 90 repetitions for all four sets), Holm et al. (2008) work matched the low-load condition to high-load, and Lixandrao et al. (2015) used an arbitrary, though commonly used, repetition protocol (3 sets of 15 repetitions). Therefore, in the Holm et al. (2008) and Lixandrao et al. (2015) studies the low-load conditions were likely stopped prior to failure, whereas the high-load conditions were at or near failure, meaning more muscle fibers would be stimulated for hypertrophy (Dankel et al., 2016). While it seems the data by Lixandrao et al. (2015) supports the need of a greater restriction pressure (80% AOP vs. 40% AOP) to augment muscle growth, it may only be working indirectly by causing fatigue to occur earlier (Ganesan et al., 2015), within the allotted goal repetitions. We argue that, were exercise performed to volitional failure, the muscle growth may have been similar across conditions. In fact, multiple experimental studies have shown that when comparing low-loads with and without BFR (Fahs et al., 2015; Farup et al., 2015), as well as low-loads and high-loads (Mitchell et al., 2012; Morton et al., 2016), when taken to failure muscle growth is similar. Overall, the current data suggests that when exercising to volitional failure the increase in muscle size is neither load nor pressure dependent, supporting a recent meta-analysis resulting in a similar conclusion (Lixandrão et al., 2018). In contrast, Lasevicius et al. (2018) conclude that there is a loading threshold between 20 and 40% 1RM that must be surpassed to optimize muscle growth. While these differences could be due to differing image analyses (the authors used separate muscle thickness images to estimate cross-sectional area), the data from Lasevicius et al. (2018) could also suggest that when equated for volume greater external loads will provide a more robust stimulus per repetition. Regardless, their finding of a need to use greater loads to maximally stimulate muscle growth is not a consistent finding and more work should be done to reconcile these differences.

As concerns exist regarding the ability to distinguish true muscle growth from muscle edema in the early phases of a training program (Damas et al., 2016), we sought to investigate the ability of the muscle to swell in response to exercise over the course of the training protocol. A previous investigation found a limit in the ability for exercise to induce muscle swelling by showing that a swollen muscle did not swell further when undergoing a second bout of exercise (Buckner et al., 2017a). We found that the exercise-induced swelling response was present at each time-point and increased over the training protocol. The increase in the swelling response across time may have been due to the increase in exercise volume across the training protocol, or perhaps the increase in muscle size, which could theoretically hold a greater volume of fluid. A previous study measuring acute exercise-induced changes in muscle thickness at the beginning and end of a training program, found a muscle swelling response at both time points (Farup et al., 2015). Thus, we believe we were measuring true muscle growth with our muscle thickness measurements, as a damaged/swollen muscle likely would not have responded to exercise-induced swelling.

### Exercise Volume

Despite the magnitude of difference in loads, 15/0 and 70/0 did not differ across most weeks. By design, repetitions were limited to 90/set, therefore, the volume was potentially limited in the 15/0 condition. We believe this generally only affected volume in the early sets as only 2 of 40 total participants eventually reached 360 repetitions during training. Although it was not required to reach volitional failure, and did not seem to augment the strength or muscle size response to very-low loads, BFR did reduce the amount of volume required to elicit adaptations in a pressure dependent manner. This may be important for a variety of populations that wish to increase muscle size and endurance but wish to limit the amount of overall work whether it be due to injury, frailty, or a simple desire to limit joint stress. In fact, very low-load resistance with 15% 1RM may more closely resemble the ability of some clinical populations rather than the more oft used 30% 1RM in low-load training protocols. Furthermore, while volume is thought to be an important training variable, the data herein suggests there may not be a dose-response relationship with respect to muscle growth, as all conditions increased muscle thickness similarly. Thus, there is likely a point where additional volume is no longer augmenting muscle growth. Future research should investigate whether a minimum volume threshold to elicit adaptation exists and whether or not that threshold differs between trained and untrained individuals.

### Limitations

Our study may have been limited by the design, requiring each participant to train unilaterally using two different conditions, potentially inducing a crossover effect between legs. However, these issues are likely minimal as both limbs were trained (MacInnis et al., 2017) and we were able to detect differences in strength changes. Our statistical analysis also helped to account for any potential issues of dependency (two conditions from each participant). BFR was based upon a percentage of resting AOP measurement, rather than quantified blood flow, thus, no assumptions can be made about the actual percent reduction in blood flow caused by the different cuff inflation pressures. While the amount of work increased most weeks, the difference in 1RM changes between conditions might have been greater had loads been progressed, however, doing so could have posed a separate set of limitations. For example, we were also attempting to determine if BFR could augment a training load that was perhaps too low for adaptation, had 15% 1RM conditions been progressed it would have limited the ability to elucidate whether adaptation was due to progressed load or BFR. Further, we felt that progressing the load in 70/0 and not the other conditions would create a greater limitation to the specific aims of the study. Also, the rest periods between sets for 15% conditions were much shorter than 70%, meaning the training density differed between conditions, likely influencing volume completed. However, rest periods used were in accordance with commonly used BFR and traditional high load protocols. Lastly, for a measure of reliability, we included a control muscle thickness site on an upper body muscle group that was not trained. Including muscle thickness measures on the leg for a time matched non-exercise control group would have been a stronger design.

### Conclusion

An increase in strength was seen in the 1RM test following high-load training only and there were no differences in conditions for isometric or isokinetic measures suggesting that increases in isotonic strength are load dependent. The increase in an unpracticed or “general strength” test did not differ due to load, suggesting that the effect of load is task specific and may not translate to other tasks well, however, this may require further research to confirm. The current data also suggest that the application of a higher BFR pressure creates a unique stimulus compared to non-restriction conditions to increase endurance. Muscle size did not depend on load, nor was it affected by the differences in volumes or restriction pressures. Given muscle size increases did not differ across conditions, despite differences in exercise volume, suggests a lack of a dose-response relationship. Furthermore, the lack of strength increase in the very low-load conditions while similar increases in muscle size were found suggests a dissociation between the two.

## Author Contributions

MJ, SB, JM, KM, SD, TA, ZB, and JL were involved with conceptualization, implementation, and data collection. MJ, JB, and JL were involved with statistical analyses. MJ, SB, JM, KM, SD, TA, ZB, JB, and JL were involved with drafting and editing the manuscript.

## Conflict of Interest Statement

The authors declare that the research was conducted in the absence of any commercial or financial relationships that could be construed as a potential conflict of interest.
